# Supplementary figures and images for: AFM characterization of early P. aeruginosa aggregates highlights emergent mechanical properties
Source: mSystems. 2025 Oct 10;10(11):e01312-25. doi: 10.1128/msystems.01312-25 (PMC12625752; doi:10.1128/msystems.01312-25)

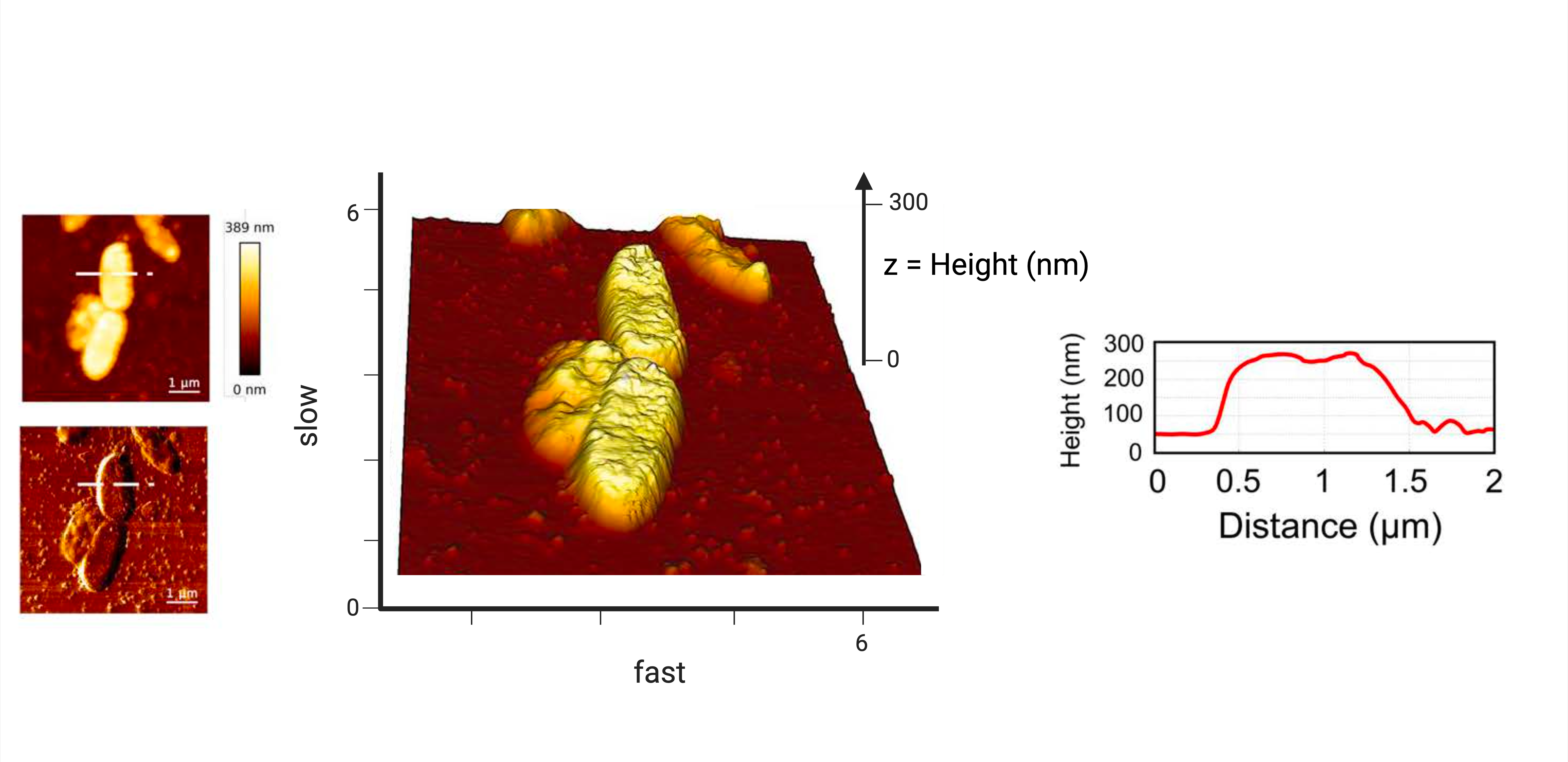

Supplement: Figure S1 — AFM cross-sectional height profile of Pseudomonas aeruginosa within an aggregate. [file msystems.01312-25-s0001.tiff]
