# Supplementary material for: AFM characterization of early P. aeruginosa aggregates highlights emergent mechanical properties
Source: mSystems. 2025 Oct 10;10(11):e01312-25. doi: 10.1128/msystems.01312-25 (PMC12625752; doi:10.1128/msystems.01312-25)
Supplement: Supplemental material — Supplemental methods and figure legend. [file msystems.01312-25-s0002.docx]

**Supplementary Information (SI)**

**Materials and methods**

**Bacterial strains and growth conditions**

*Pseudomonas aeruginosa* PAO1 wild type carrying the plasmid pMRP9-1, expressing GFP, was cultured in standard lab media (LB) from frozen stock overnight at 37°C with shaking (200 rpm)^1, 2^. Cells were back diluted 1:20 in fresh LB and grown to log-phase (~3 hours), then washed with PBS (pH7.0) before inoculation^2^. For aggregates, SCFM2 containing mucin (SCFM2 + mucin) (Porcine mucin, 250 mg) was prepared as previously described^2, 3^ (obtained from SynthBiome). For planktonic cultures, SCFM2 without mucin (SCFM2 - mucin) was prepared identically but with the exclusion of mucin^2^. Washed *Pa* cells were inoculated into both media at approximately 10^5^ cells per mL (0.05 OD^600^) and incubated without shaking at 37°C for 4 hours to allow for aggregate formation^2, 4^.

**Atomic Force Microscopy (AFM) analysis of *P. aeruginosa* aggregates and planktonic cells**

**Sample preparation:** For both growth conditions (SCFM2 -/+ mucin), 4-hour cultures were diluted 1:20 in PBS to create a ‘low density culture’. A hydrophobic pen was used to create a liquid repellent area (circular) on poly-L-lysine coated microscope slides (Fisher Scientific) to which 20 μL of sample was pipetted into the center. Throughout sessions, 100-200 μL of molecular grade water was supplemented as needed for hydration and to improve resolution of individual cells and/or aggregates.

**Atomic Force Microscopy:** A JPK Nanowizard PURE atomic force microscope (Bruker Nano, Berlin, Germany) mounted on an inverted epifluorescence Zeiss Axiovert 200 M microscope (Carl Zeiss Microscopy, Göttingen, Germany) was utilized for imaging and mechanical characterization of *P. aeruginosa* samples at the micro- to nanoscale. The samples were imaged and mechanically tested following the developed protocols^5^. Multiple images were captured at each location on each sample using contact mode imaging. The maximum lateral scan focused on regions measuring 50 μm × 50 μm, with a scanning rate set to 1 Hz. Reduced scan areas were then selected to obtain detailed structural information of the samples. MSNL-10 silicon nitride cantilevers (Bruker, Mannheim, Germany) with a spring constant of 0.01-0.1 N/m and a nominal tip radius of 2 nm were used for imaging. To ensure that the same region of interest was analyzed following probe exchange, we used the motorized XY stage of the JPK Nanowizard system. The stage allows precise, computer-controlled repositioning to previously defined coordinates, which are stored by the software during the initial high-resolution scan. After changing to the blunt probe for force spectroscopy, the stage was guided back to the exact same coordinates. To confirm correct alignment, topographical landmarks within the selected region were cross-checked before indentation measurements were performed. This approach ensured reproducibility of measurements from the same imaged area without loss of positional accuracy. For indentation measurements, Biosphere Au Reflex (CONT-Au) cantilevers (Nanotools USA LLC, Henderson, NV) with a nominal spring constant of 0.2 N/m, a length of 450 μm, and a nominal resonance frequency of 13 kHz in air, equipped with integrated spherical tips of radii 100 nm and 2 μm (±10%), were employed ^6, 7^. All measurements were conducted in a wet environment with the sample immersed in PBS. The indentation rate was set to 2 μm/s. Before each test, the deflection sensitivity of the cantilever was calibrated by engaging it on a clean glass slide in a wet state ^6, 7^. The exact spring constant of the cantilever was calibrated using thermal noise fluctuations in air ^5^. This was done by fitting the first free resonance peak of the cantilever to that of a simple harmonic oscillator using the JPK software ^5^. The software employs various models, such as Hertz and Sneddon contact mechanics, to extract the elastic modulus from force-displacement curves obtained during indentation tests ^8^. The elastic modulus was obtained from the Hertzian contact model ^8^. In this model, the contact radius a is related to the indenting force F through

$$a=\left( \frac{3RF\left( 1-\nu^{2} \right)}{4E} \right)^{1/3}$$

with R being the radius of the spherical tip, and $\nu$ and E being the Poisson’s ratio and elastic modulus of the sample, respectively ^8^. The indentation depth $\delta$ is expressed in terms of the contact radius as

$$\delta=\frac{a^{2}}{R}=\left( \frac{{9F}^{2}\left( 1-\nu^{2} \right)^{2}}{16RE^{2}} \right)^{1/3}$$

The JPK data processing software was used to analyze the indentation data.

**Figure S1: AFM cross-sectional height profile of *Pseudomonas aeruginosa* within an aggregate.**

Representative AFM height images and corresponding cross-sectional analysis demonstrate the vertical dimension of individual *P. aeruginosa* cells within an early-stage aggregate (4 hours growth). The 3D topography (center) and line profile (right) indicate an average cell height of ~300 nm, consistent with previously reported dimensions of hydrated *P. aeruginosa* cells in aggregates. Additional AFM height maps (left) show top-view scans with cross-section lines used for profiling. Together, these images confirm that aggregates represent physiological *in-situ* dimensions during AFM imaging and analysis.

**References**

1. Davies DG, Parsek MR, Pearson JP, Iglewski BH, Costerton JW, Greenberg EP. The Involvement of Cell-to-Cell Signals in the Development of a Bacterial Biofilm. Science. 1998-4-10;280(5361). doi: 10.1126/science.280.5361.295.

2. Gannon AD, Matlack J, Darch SE. Exploring aggregation genes in a *P. aeruginosa* chronic infection model. Journal of Bacteriology. 2024-12-11. doi: 10.1128/jb.00429-24.

3. Gannon AD, Darch SE. Tools for the Real-Time Assessment of a *Pseudomonas aeruginosa* Infection Model: 1940-087X; 2021. e62420 p.

4. Darch SE, Kragh KN, Abbott EA, Bjarnsholt T, Bull JJ, Whiteley M. Phage Inhibit Pathogen Dissemination by Targeting Bacterial Migrants in a Chronic Infection Model. mBio. 2017-4-4;8(2). doi: 10.1128/mbio.00240-17.

5. Asgari M, Brulé V, Western TL, Pasini D. Nano-indentation reveals a potential role for gradients of cell wall stiffness in directional movement of the resurrection plant Selaginella lepidophylla. Scientific Reports. 2020;10(1):506. doi: 10.1038/s41598-019-57365-z.

6. Asgari M, Latifi N, Giovanniello F, Espinosa HD, Amabili M. Revealing Layer‐Specific Ultrastructure and Nanomechanics of Fibrillar Collagen in Human Aorta via Atomic Force Microscopy Testing: Implications on Tissue Mechanics at Macroscopic Scale. Advanced NanoBiomed Research. 2022;2(5):2100159.

7. Asgari M, Mirzarazi E, Vali H, Frisina RD, Espinosa HD. Ultrastructural viscoelastic behavior of collagen identified by AFM nano-dynamic mechanical analysis. bioRxiv. 2024:2024.10. 19.619231.

8. Hertz H. Ueber die Berührung fester elastischer Körper1882.
